# The hierarchical age–period–cohort model: Why does it find the results that it finds?

**DOI:** 10.1007/s11135-017-0488-5

**Published:** 2017-02-24

**Authors:** Andrew Bell, Kelvyn Jones

**Affiliations:** 1 Sheffield Methods Institute, University of Sheffield, ICOSS Building, 219 Portobello, Sheffield, S1 4DP UK; 2 School of Geographical Sciences, University of Bristol, University Road, Bristol, BS8 1SS UK

**Keywords:** Age–period–cohort, Hierarchical age period cohort model, Obesity, MCMC, Identification, Multilevel modelling

## Abstract

It is claimed the hierarchical-age–period–cohort (HAPC) model solves the age–period–cohort (APC) identification problem. However, this is debateable; simulations show situations where the model produces incorrect results, countered by proponents of the model arguing those simulations are not relevant to real-life scenarios. This paper moves beyond questioning whether the HAPC model works, to why it produces the results it does. We argue HAPC estimates are the result not of the distinctive substantive APC processes occurring in the dataset, but are primarily an artefact of the data structure—that is, the way the data has been collected. Were the data collected differently, the results produced would be different. This is illustrated both with simulations and real data, the latter by taking a variety of samples from the National Health Interview Survey (NHIS) data used by Reither et al. (Soc Sci Med 69(10):1439–1448, 2009) in their HAPC study of obesity. When a sample based on a small range of cohorts is taken, such that the period range is much greater than the cohort range, the results produced are very different to those produced when cohort groups span a much wider range than periods, as is structurally the case with repeated cross-sectional data. The paper also addresses the latest defence of the HAPC model by its proponents (Reither et al. in Soc Sci Med 145:125–128, 2015a). The results lend further support to the view that the HAPC model is not able to accurately discern APC effects, and should be used with caution when there appear to be period or cohort near-linear trends.

**Electronic supplementary material:**

The online version of this article (doi:10.1007/s11135-017-0488-5) contains supplementary material, which is available to authorized users.

## Introduction

The hierarchical age period cohort (HAPC) model has, like every age–period–cohort (APC) model that has been proposed in the last 50 years, received a mixed reception since it was first outlined in 2006 (Yang and Land 2006). Whilst it has been taken up enthusiastically in parts of the social and medical sciences, the ability of the model to produce meaningful statistics has also been disputed. This is because it is, apparently, attempting to do the impossible (Bell and Jones 2013): separating age, period and birth cohort effects, including linear effects if they are present. Many, including us, have argued that it doesn’t work, and used simulations to demonstrate the situations in which this is the case (Luo and Hodges 2016; Bell and Jones 2014a). The inventors of the model and others have responded that simulations are an inappropriate method for assessing the importance of APC methods (Reither et al. 2015a). This paper can be considered the next entry in this continuing debate.

This debate is an important one. Many applied researchers now see the HAPC model as the “standard way of analysing generational effects” (Linek and Petrúšek 2016, p. 82), even whilst acknowledging the critics of the method. Whilst the methodological questions remain open, such judgements will continue to be made. This is a problem if, as we believe, the model does not function as its proponents suggest it does, and can produce highly misleading results. The debate also mirrors and complements that taking place elsewhere regarding another APC model called the Intrinsic Estimator (see Pelzer et al. 2015; Te Grotenhuis et al. 2016; Yang and Land 2013b; Luo 2013a, b; Luo et al. 2016).

In the latest rejoinder on this subject to our earlier critique, Reither et al. (2015a) left a number of unanswered questions, and we hope to be able to give our answers to those questions here. However, the key focus of this paper lies in making an argument not just that the HAPC model sometimes doesn’t work, but also in giving a reason why the model produces the results that it does. Moreover, this paper moves the debate around the HAPC model beyond simulations, towards the analysis of real data. This is not to say that we consider previous simulation studies worthless; rather that we believe that the case presented by simulations is already rather convincing, and in showing that similar results occur in real-life data, it lends credence to the argument that those simulations did indeed produce results that are indicative of real-world scenarios, despite Reither et al.’s (2015a) claims to the contrary. We will show, using both real and simulated data, that the results produced are the result not of substantive processes at hand, but an artefact of the structure of the data being analysed. When taking different samples from the *same* given real-life dataset, you can get different results depending on how you select your sample. This gives insight into why the HAPC model produces the results that it does—simulations have already shown that often the results they produce are incorrect, but have not thus far given any insight as to why.

Readers might feel that, in furthering the critique of the HAPC model, this paper is simply ‘flogging a dead horse’, given the existing critiques by many separate researchers (see Table 1). We disagree and contend that this paper makes three important contributions. First, practitioners are still using the HAPC model, and this paper we hope encourages readers to be critical of the model, and not take the latest rejoinder (Reither et al. 2015a) as the final word on the subject. Second, it offers useful insight to methodologists in understanding how statistical models generally, and multilevel models in particular, behave in the presence of exact collinearity in the random effects. Third, in comparing simulated results to real data, it shows the value that simulation can offer, in contrast to Reither et al. (2015a) who seem to argue its use is problematic because it is, in a sense, synthetic and therefore unrealistic.

**Table 1:** Key papers (and arguments made) in the debate around the HAPC model

Paper	Argument
Yang (2006)	Argues the HAPC model can be used in a Bayesian framework. Uses real data on verbal test scores, and simulations (note that the latter’s DGPs have only independent and identically distributed Normal random variation to generate the period and cohort effects). [51 cites in Google Scholar as of 8th Feb 2017]
Yang and Land (2006)	Argues the treatment of age as quadratic in the HAPC model solves the identification problem. Example using real data on verbal test scores [233]
Yang and Land (2008)	Uses the Hausman test (on multiple parameters) to test if fixed or random effects should be used for the period and cohort terms. Example using real data on verbal test scores [237]
Yang and Land (2013a)	Book argues the different treatment of age (fixed) and period/cohort (random) “completely avoids” (p. 70) the identification problem. Uses various real data sources to illustrate this [132]
Bell and Jones (2014a)	Argues with simulations that the HAPC model is not good at recovering DGPs in the presence of linear effects
Bell and Jones (2014c)	Argues that results can be reproduced using a completely different DGP do that suggested by those results
Reither et al. (2015b)	Argues that linear effects do not occur in real-life data, and thus that the model works for real data (this is illustrated, ironically, with simulations)
Bell and Jones (2015b)	Argues with simulations that even when the DGP does not include exactly linear effects, the HAPC model does not work
Reither et al. (2015b)	Argues that model fit statistics, and descriptive and modelled graphics, should be used to judge whether the HAPC model is appropriate for use
Luo and Hodges (2016)	Argues that grouping cohorts in different ways can produce arbitrarily different results, using simulations
O’Brien (2016)	Demonstrates why treating one or more of APC as random effects allows models to be identified, but shows that the solution that is arrived at is an artefact of the way the log likelihood is maximized
Fienberg et al. (2015)	Responding to a positive book review they contend that “Yang and Land’s approaches really are no different from previous attempts to resolve the APC identification problem insofar as they impose constraints on the estimated age, period, or cohort effects; the constraints are simply hidden in the technical details of their methodology” (p. 457)

Papers with Reither or Yang as first author are proponents of the model, others are for the most part critical of it

A somewhat parallel debate also exists on Yang and Land’s Intrinsic estimator as a means of tackling the problem (see Pelzer et al. 2015; Te Grotenhuis et al. 2016; Yang and Land 2013b; Luo 2013a, b; Luo et al. 2016)

This paper begins with a brief discussion of the APC identification problem, before outlining our explanation for why the HAPC model produces the results that it does under different data scenarios. We argue that it is the range of the periods and cohorts set by the data structure, rather than any substantive processes, that drives the results that are found. This is illustrated first with simulations and second by attempting to replicate Reither and colleagues’s (2009) study of APC effects on obesity in the United States. Regarding the latter, whilst we were able to replicate the study using the full data (including additional data up to and including 2014) we show that we get different results when we take particular samples of the data, with Reither et al.’s results not replicated when data is sampled based on a narrow range of cohorts. By way of a coda to the article, we rebut the key points made by Reither et al. (2015a) in their most recent rejoinder—particularly regarding the use of model fit statistics, and the use of both descriptive and modelled APC graphical trends to test whether the use of the HAPC model is appropriate. The paper finishes with a summary of the arguments in favour of the HAPC model so far, and suggestions for what substantive researchers interested in APC processes should do in the light of these criticisms.

## The key critique of the HAPC model

The debate around the HAPC model has been extensive, and the key contributions to it are summarised in Table 1 for readers to consider themselves. The problem that the model is trying to address is that age, period (year) and cohort (year of birth) are linearly related such that age = period − cohort. This is a problem if any of age, period or cohort are linearly related to a given outcome, since different linear combinations of APC can produce identical outcomes.

For us, the key critique of the HAPC model lies in its inability to accurately represent data generating processes (DGPs) in simulation. In particular, we have shown (Bell and Jones 2014c) that results that have been found in previous work in fact could have resulted from an entirely different DGP. This has been shown, both with linear and non-linear relations (Bell and Jones 2015b); a non-linear relationship with an outcome does not mean there isn’t also a linear relation that could cause a problems in attempting to uncover true APC trends, even when no linear effect is included in the DGP.

## What drives the HAPC model to period trends?

All of the above are in our view good reasons why the arguments in favour of the HAPC model should be viewed with scepticism. However, there remain a number of questions that critics of the HAPC model have not yet answered. In particular, why is it that that the HAPC model finds the results that it finds? Simulations have shown that the HAPC model tends to favour period effects over cohort effects, but that this is not consistent when cohorts are grouped (Bell and Jones 2014a). Yet there has been no discussion in the literature to our knowledge as to why that pattern occurs.

Here we present an argument that lies in the imposed structure of the data being analysed. The HAPC model is designed for repeated cross sectional data, where a sample is taken across a number of years, and so this data can be represented in a rectangular age-by-period table. Similarly, panel data (for which the HAPC model has been adapted—see Suzuki 2012) can also be represented in such a format. The result of such data is that cohorts—represented by the diagonals in an age-by-period table and measured by year of birth—span a wider range of years than periods. Taking the data used by Reither et al. (2009) in their analysis of obesity, periods span the time period 1976–2002—a range of 26 years, whilst birth cohorts (measured by the year of birth) span the years 1890–1985—a range of 95 years.

In the HAPC model, the estimation method, whether frequentist (e.g. maximum likelihood) or Bayesian (e.g. MCMC), aims to minimise the amount of unexplained variation in the model (O’Brien 2016). In the HAPC model, the period and cohort random effects are considered, at least in part, unexplained, since they are in the random part of the model, whilst the age trend is considered explained since it is in the fixed part. The model will thus apportion variation to the trends in such a way that makes those unexplained components as small as possible, regardless of its effect on the explained part (the age parameter estimates).

**Fig. 1:**
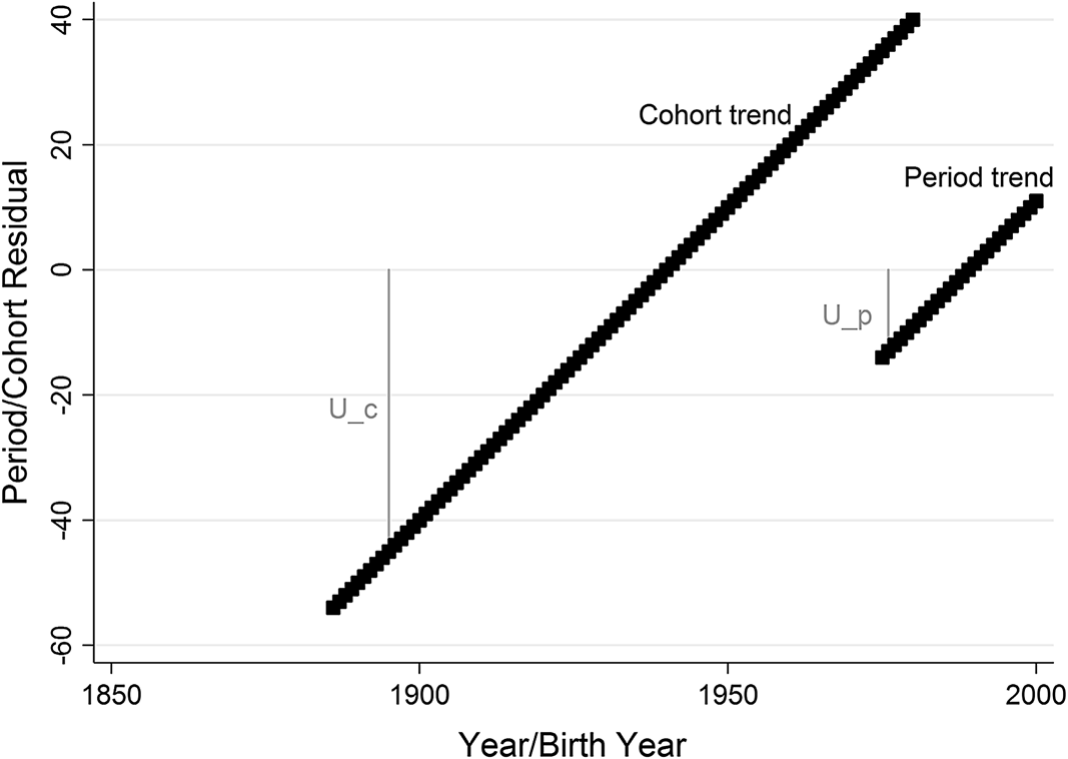
Hypothetical period and cohort trends with a slope of one, for data with the structure of that used in Reither et al. (2009). As can be seen, the cohort trend of necessity produces much more extreme residual values than the period trend, despite both having the same slope value

Imagine, for example, the true DGP of a model consists only of a linear cohort trend with a slope of 1. In this case, the HAPC model could assign the linear trend (correctly) to the cohort residuals, or it can apply it to the period trend, with an additional age trend in the opposite direction estimated in the fixed part of the model, cancelling it out (since cohort = period − age). Adding a slope to the age trend does not in any way increase or decrease the unexplained variance, so the question is which of the periods or cohorts increases the unexplained variance the most. The answer is the cohorts, because it has a wider range. The random effects attached to the very new and very old cohorts (U_c in Fig. 1) will be much bigger than the equivalent random effects for periods (U_p in Fig. 1), because a trend with a slope of 1 that spans 95 years (the range of cohorts) will reach much higher and lower values than a slope with the same gradient that spans 26 years (the range of periods), as shown clearly in Fig. 1. In Bayesian estimation, the larger variance of the cohorts would also make the effective number of parameters greater (a wider spread of cohorts results in those cohort residuals counting as more effective parameters—Spiegelhalter et al. 2002).

Of course, a true DGP is unlikely to be as simple as a single linear effect. But if there is a single linear or near-linear effect as part of the DGP, the model will assign that trend in such a way that reduces the unexplained variance, and so, all other things being equal, will place it with period effects.

As stated previously, grouping has an effect on this, making the direction of the effect assignment more unpredictable. Whilst grouping does not affect the range spanned by the cohorts, it would affect the number of cohort groups. On one hand, grouping cohorts makes the measurement of cohorts less precise and so make the fit of the cohorts to the data worse, which might lead the model to ‘favour’ during estimation the more finely grouped periods for a trend. On the other hand, if there are fewer groups, there are fewer random effects and so either fewer degrees of freedom consumed (in a Bayesian model) or smaller penalties to the log-likelihood (O’Brien 2016). Whilst on average the results seem to fit the period solution on average, there is more variation around this in possible results from the same DGP (as shown by the simulations in Bell and Jones 2014a), and differently grouping the same dataset will produce fundamentally different results (Luo and Hodges 2016).

The key point is that the data structure, and thus the tendencies towards periods described here, are not the result of any real-world substantive process and thus their influence on the results is a statistical artefact. One could, instead, collect data by cohorts; that is, follow a large number of birth cohorts through their lives. The result would be a rectangular age-by-cohort table, with periods along the diagonals. In this situation, there would be a much wider range of periods than cohorts, and the model would tend to assign trends to cohorts instead of periods. This change would not be substantive—it would merely be a result of the data structure.

## Simulations

In order to test this, we simulated some data that was collected (1) as if selected by periods, and (2) as if selected by cohorts. The DGP for both datasets is as follows:

1 $$\begin{aligned} {\text{Y}} & = 1 + \left( {0.1*{\text{Age}}} \right) + \left( { - 0.005*{\text{Age}}^{2} } \right) + \left( { - 0.01*{\text{Year}}} \right) + \left( { - 0.002*{\text{Year}}^{2} } \right) \\ & \quad + {\text{u}}_{\text{C}} + {\text{u}}_{\text{p}} + {\text{e}}_{\text{i}} \quad {\text{e}}_{\text{i}} \sim\,{\text{N}}\left( {0,4} \right),{\text{u}}_{\text{c}} \sim\,{\text{N}}\left( {0,1} \right), {\text{u}}_{\text{p}} \sim\,{\text{N}}\left( {0,1} \right) \\ \end{aligned}$$

where e_i_ is the level 1 residuals, Normally distributed with a variance of 4, and u_c_ and u_p_ are the cohort group and period residuals, each randomly Normally distributed with a variance of 1. Age and Year are centered on 40 and 1990 respectively, and cohorts grouped into 3 year intervals. This data was generated (1) for samples of individuals aged 20–60 taken in years 1990–2010, and (2) for individuals born between 1930 and 1965, and sampled between age 20 and 60. Thus, in the former cohorts spanned a wider range than periods, and in the latter, the situation is reversed, but in both cases the underlying data generating process is exactly the same; the same age and period linear and quadratic effects and the cohort and the period differences are generated to have the same variance. The datasets, each with 20,000 observations, were fitted to the HAPC model:

2 $$\begin{aligned} y_{{i\left( {j_{1} j_{2} } \right)}} & = \beta_{{0j_{1} j_{2} }} + \beta_{1} Age_{{i\left( {j_{1} j_{2} } \right)}} + \beta_{2} Age_{{i\left( {j_{1} j_{2} } \right)}}^{2} + e_{{i\left( {j_{1} j_{2} } \right)}} \\ \beta_{{0j_{1} j_{2} }} & = \beta_{0} + u_{{1j_{1} }}+ \,u_{{2j_{2} }} \\ & \quad e_{{i\left( {j_{1} j_{2} } \right)}} \sim\,N\left( {0,\sigma_{e}^{2} } \right), \,u_{{1j_{1} }} \sim\,N\left( {0,\sigma_{u1}^{2} } \right), \,u_{{2j_{2} }} \sim\,N\left( {0,\sigma_{u2}^{2} } \right) \\ \end{aligned}$$

Here, i represents individual observations, j_1_ represents cohort groups, and j_2_ represents years. This model is run using the same 3-year cohort intervals, using MLwiN 2.36 (Rasbash et al. 2011) with the runmlwin command (Leckie and Charlton 2013) in Stata, with a 100,000 iteration chain length, a 5000 iteration burn-in, and true starting values (in other words, we are being as kind to the model as possible, by actually giving it the true answers as a starting point).

**Fig. 2:**
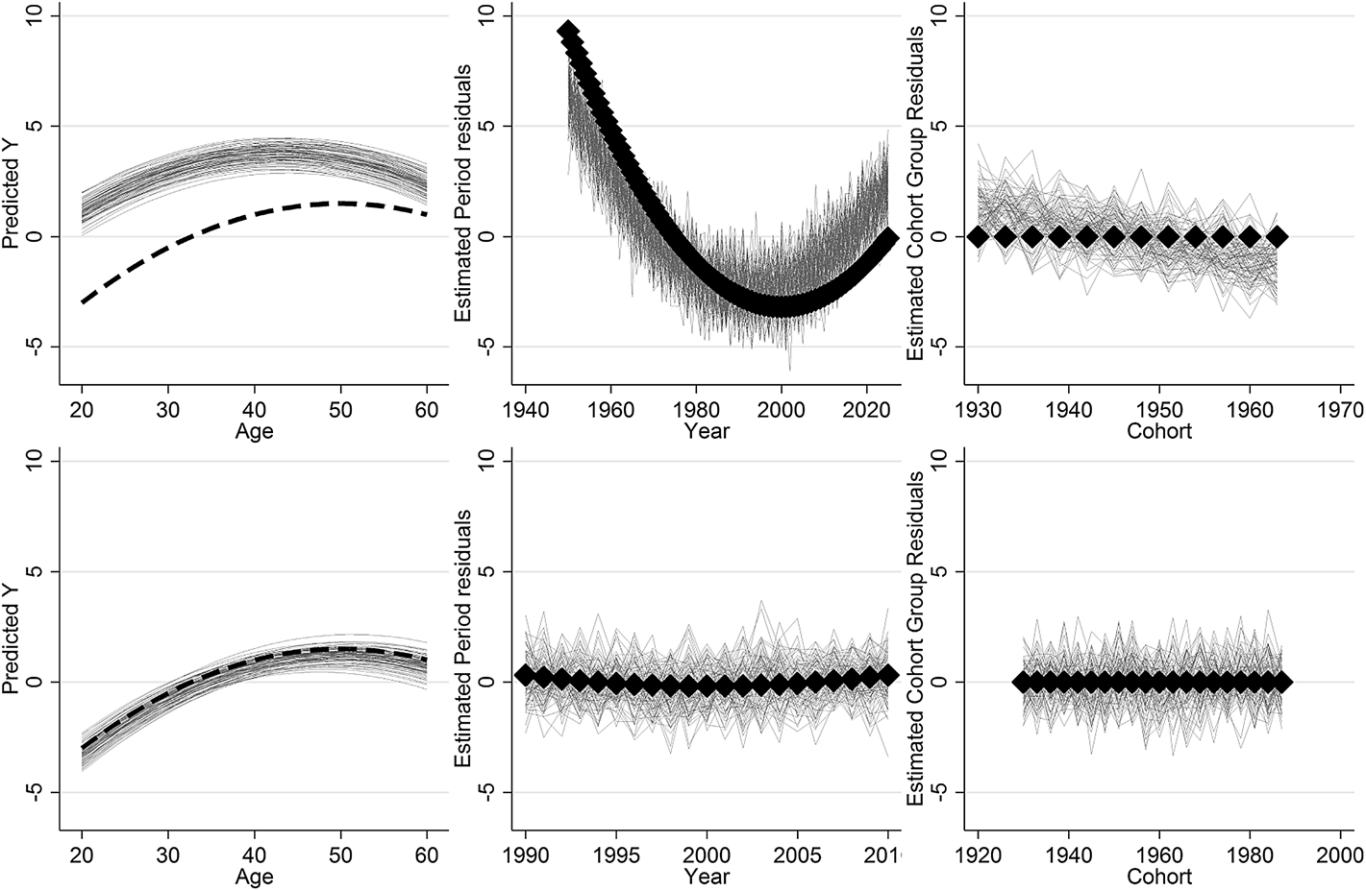
Simulation results from the DGP in Eq. (1), with results (*thin grey lines*) compared to the truth (*large back lines/points*). *Row 1*: age-by-cohort data; *row 2*: age-by-period data

The results are shown in Fig. 2. When the data is in the form of an age-by-cohort table (row 1), the model incorrectly assigns a linear trend to cohorts, and consequently misestimates the age and period trends. When the data is in the form of an age-by-period table (row 2) the model assigns the trend to periods, and so accurately estimates all three trends, but of course it would have found this period trend were the true linear trend a cohort effect, as shown previously (Bell and Jones 2014a). This is the case even though there is significant non-linear variation in the DGPs—the linear component is still reassigned in the way suggested above. This provides compelling evidence that it is the data structure that is driving the results that are found. It is only the data structure that changes between these two scenarios; the data generating process has not been changed.

## Real data

Reither et al. (2015a) argue that using simulated data “is not a productive way to advance the discussion” (p. 125). Whilst we do not agree with this, we understand that readers may not be convinced by evidence based on data that is in some sense not real. Because of this, we have additionally tested our explanation with real data. This is not easy to do. Whilst one could collect data by cohorts, this would be extremely costly in time and money (to get a full range of age groups, you would have to measure each cohort of people every year for their entire lives). Instead, we take a real-life dataset and take a number of samples that mimic the properties of the data collected by periods and cohorts as described above.

We use the National Health Interview Survey dataset (National Center for Health Statistics 2004) used by Reither et al. (2009) in their study of obesity. We were able to extend Reither et al.’s analysis by including data up to and including 2014. The only other difference between our study’s analyses and that of Reither et al. was that we were unable to replicate exactly the adjustment they performed on their outcome variable, the body mass index (BMI) that measures obesity (whilst we contacted the authors in the hope of replicating this adjustment exactly, no reply was forthcoming). This adjustment is necessary due to (1) a change in the way obesity was measured in 1997 to exclude proxy-reporting, and (2) a generally-observed increase in downward-bias that appears to have been present over the study period (Reither et al. 2009 p. 1441). Instead, we used Fig. 1 in Reither et al. (2009) to attempt an approximate adjustment to the measure of obesity. Specifically, measured BMI was adjusted by adding on 0.5 + 0.03 * (year−1976) for years before 1997, and by adding on 0.75 + 0.03*(year−1976) for years 1997 and onwards. Obesity was then defined as those with an adjusted BMI of 30 or more. The results do not appear to be affected by this adjustment, and, when using the full dataset (both up to 2002 and including data up to 2014), we were able to replicate Reither et al.’s results (see Fig. 4), suggesting the adjustment is good enough to make the methodological point at hand in this paper.

**Fig. 3:**
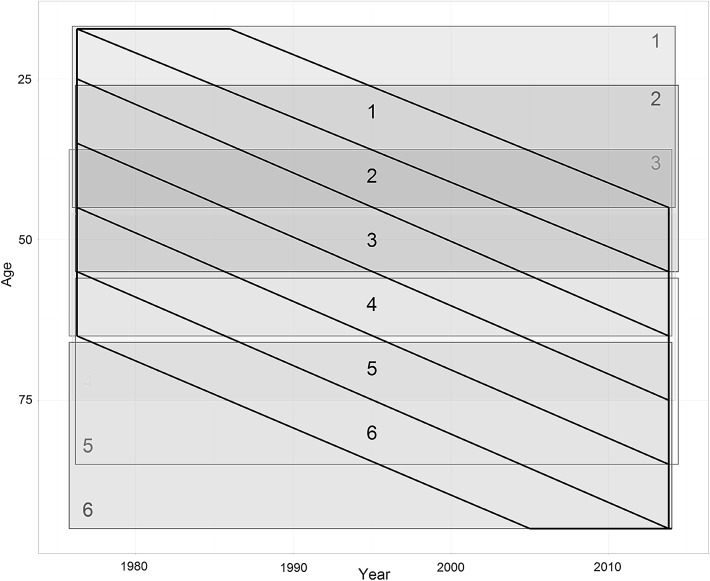
Age-by-period representation of the full NHIS dataset, with the 12 samples taken shown. Samples defined by cohorts (10 years) are in *black*; samples defined by age (30 years) are *coloured*. (Color figure online)

In order to evaluate whether the results are different when data has different structures, we take a number of different samples from the data; these are represented in Fig. 3. First, we take a number of samples each of which are based on cohorts (the black boxes in Fig. 3). For each such sample, the entire range of years (39) is included, but the range of cohorts is limited to a 10-year birth cohort span (as shown in black in Fig. 3). The reason for choosing 10-years as the range for cohorts is because it makes it approximately one-quarter of the range of periods, which is similar to the range of periods in relation to the range of cohorts in standard repeated cross-sectional data (including the ranges of periods and cohorts in Reither et al.’s original study).

Second, we took a number of samples that were based on age (with an arbitrarily chosen range of 30). In this case, the range of cohorts is still greater than the range of periods, but to a lesser extent than in the overall sample. The purpose of these models was to check whether the results found by HAPC differed across the age range, which might explain any differences found in the cohort-sampled models. These samples are represented by the coloured boxes in Fig. 3.

The HAPC model was applied to both the full dataset and the samples outlined here. This was done both without any grouping, and by including grouping for year groups (note, with only a 10 year span of birth cohorts in some models, grouping of cohorts was not possible in the cohort-selected samples). All models were run using MCMC estimation (Browne 2009), with 500,000 iterations and a 50,000 iteration burn-in, with hierarchical centering to speed up convergence and models checked for convergence using visual diagnostics. Models were again run in MLwiN 2.36 (Rasbash et al. 2009) using the runmlwin command in Stata (Leckie and Charlton 2013).

## Real-life data results

**Fig. 4:**
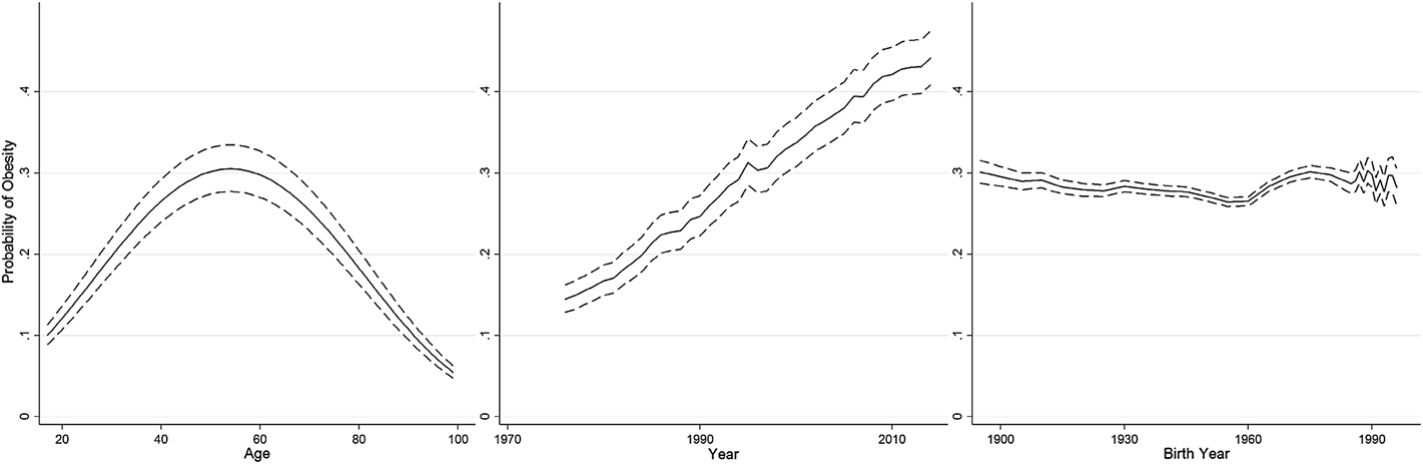
Replication of Reither et al. (2009), with data included up to 2014. Model uses 5-year birth cohort groups (the results are the same with no grouping, and with 3-year period groups)

When using the full NHIS data, we were able to replicate the results found by Reither et al. (2009) as shown in Fig. 4: an inverse U-shape in the age trend, an approximately linear period trend, and an approximately flat cohort trend (with a slightly higher obesity on cohorts born after around 1970). This result occurs regardless of whether periods and cohorts are ungrouped, periods are grouped, or cohorts are grouped, and whether the data used includes years after 2002 or not.

**Fig. 5:**
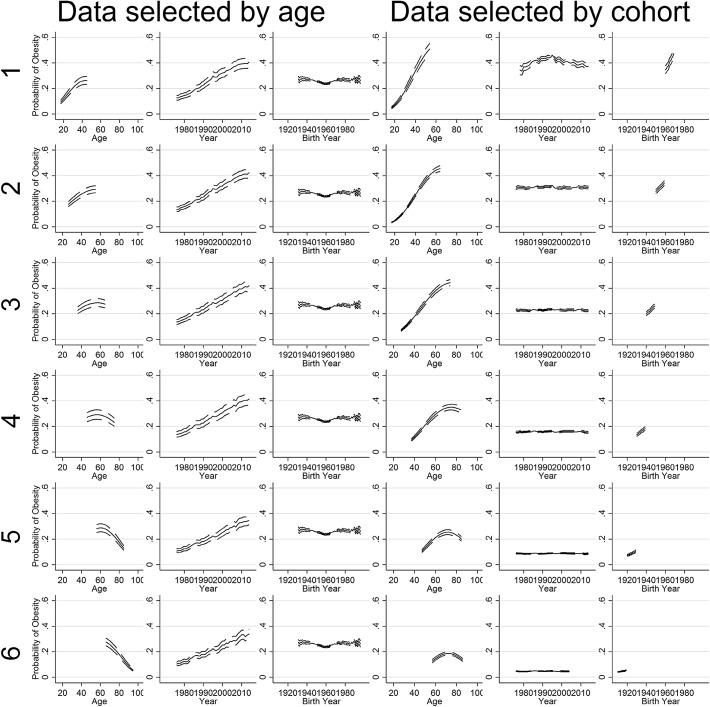
Results for models without grouping in the random effects

**Fig. 6:**
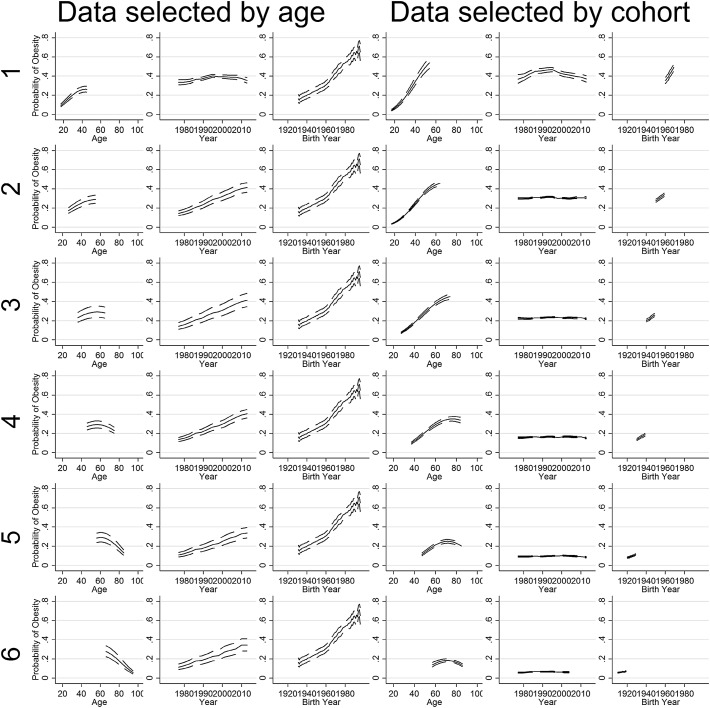
Results with periods grouped into 3-year time intervals

When data is selected by age, the results are less consistent, as shown in Figs. 5 and 6. When data are ungrouped (Fig. 5, columns 1–3), the results also match those of Reither et al. consistently, with a near-linear period trend and a flat cohort trend. However when the model is run with years grouped into 3-year intervals (Fig. 6, columns 1–3), the result is different: in general the temporal trend is split between periods and cohorts, except in one case when the entire trend is in cohorts. Note when the model is run for these data samples with cohorts grouped into 5-year intervals (not shown), the results mostly match those of (Reither et al. 2009), except in one instance where a near-linear cohort trend (and a flat period trend) is found.

When data is selected by period, the results are consistent: a near-linear positive cohort trend and a flat period trend are found, regardless of whether years or cohorts are grouped or not. That is, Reither et al.’s results are not replicated in these scenarios.

These results are entirely consistent with previous simulations (Bell and Jones 2014a, c, 2015b), the simulations presented earlier in this paper, and with the logic we have spelled out in this paper. When cohorts span a wider range than periods, as in standard repeated cross-sectional data, the trend tends towards periods, although this is complex when periods or cohorts are grouped. In contrast, when there are more periods than cohorts (i.e data is selected by cohorts), the result consistently finds the opposite result, with the trend being found in cohorts. Finally, as expected, the results are less consistent when grouping is used in one of the sets of random effects. To be clear, this is not to say any particular result found here is right or wrong with regard to obesity; rather that the data and the HAPC model alone give us no indication as to which pattern is correct.

## Responses to Reither et al. (2015a)

**Fig. 7:**
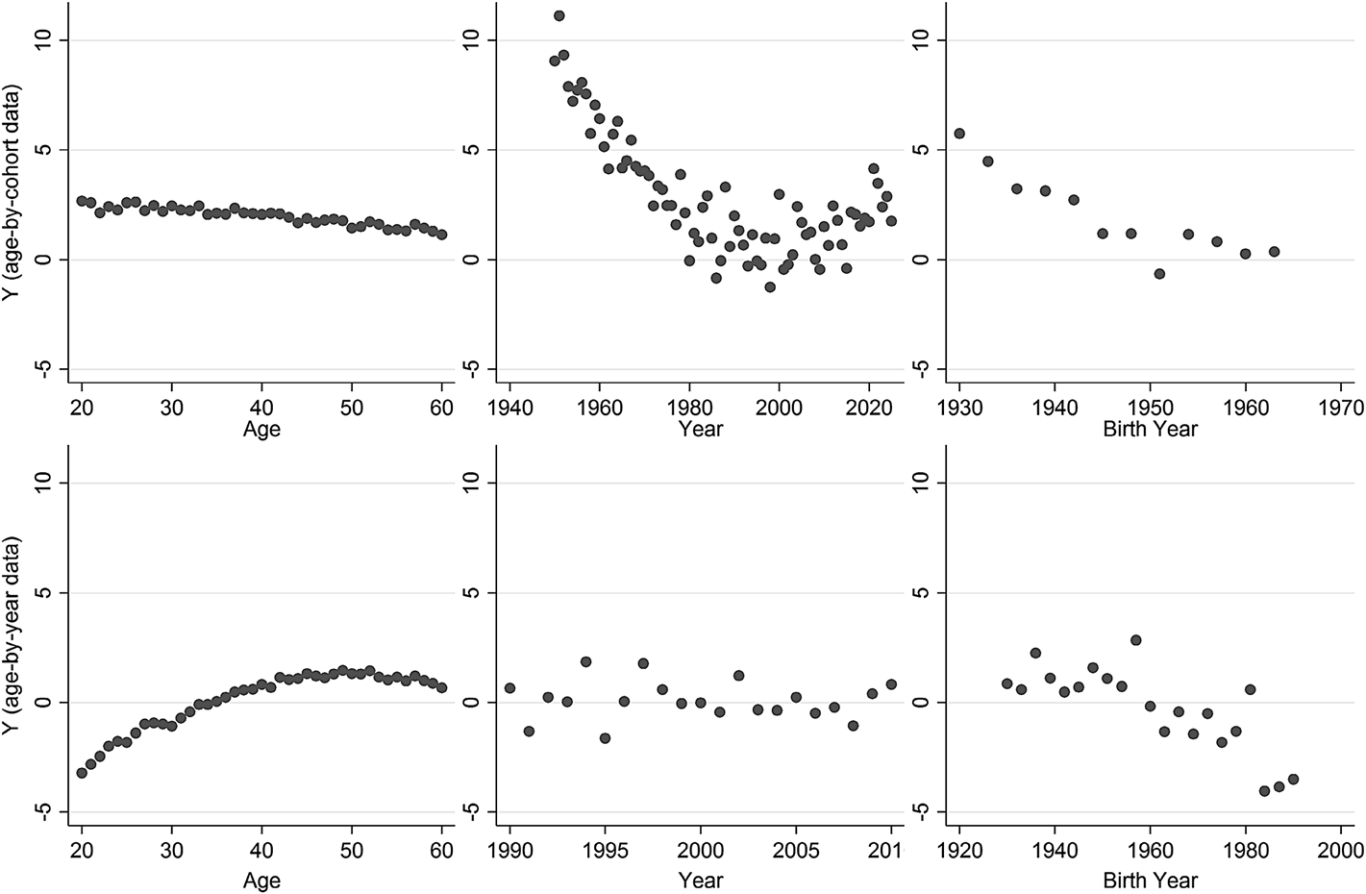
Descriptive APC plots for an example dataset used in the simulations here. *Row 1*: age-by-cohort data; *row 2*: age-by-period data

By way of a coda to this article, we now respond to the points made by Reither et al. (2015a). In their article, they list a number of criteria for the use of the HAPC model, which the simulated data in Bell and Jones (2015b) do not fulfil. We address each of these criteria below. However, it should be noted that the criteria are completely met by the simulations run in Bell and Jones (2014a), and in the simulations in this article, above (see Table 2 for model fit statistics, Fig. 7 for descriptive APC plots, and Fig. 2 for modelled APC plots). In each situation, the HAPC model again failed to recover the true parameters. In any case, we consider each of their recommended criteria below.

**Table 2:** Model fit statistics for an example dataset used in the simulations here (based on the models used in Reither et al. 2015a)

Model	*N*	Age-by-cohort data			Age-by-period data		
		*df*	AIC	BIC	*df*	AIC	BIC
APC	20,000	89	84,732.58	85,435.99	43	84,707.87	85,047.72
AP	20,000	78	86,179.2	86,795.68	23	88,936.46	89,118.24
AC	20,000	14	93,592	93,702.65	23	88,158.13	88,339.91
A	20,000	3	10,2007.3	102,031	3	91,701.97	91,725.68
P	20,000	76	91,382.66	91,983.32	21	94,676.62	94,842.6
C	20,000	12	94,035.69	94,130.53	21	89,708.2	89,874.18
AC (both quadratic)	20,000	4	94,427.87	94,459.49	5	91,686.3	91,725.82

### The use of model fit statistics

In their previous commentary, Reither et al. (2015b) argued that fit statistics should be used to assess whether the full APC model is appropriate. This point is reiterated in their latest rejoinder (Reither et al. 2015a) where they make a number of points that for us are contentious.

First they present the results of model fit statistics applied to simulated datasets. These show that “*In no instance do these model selection statistics point to a fully three*-*dimensional data structure”* (Reither et al. 2015a, p. 127, emphasis their own). This is true; however the test that they are applying is not really relevant to whether the HAPC model should be used. They are testing a model with linear and quadratic age, and period and cohort dummy fixed effects (the ‘age + period + cohort’ model), against a model with linear and quadratic age and cohort trends (the ‘age + cohort (both quadratic)’ model). The latter has just 5 degrees of freedom, whilst the former has 46, so given the true data generating process (DGP) is primarily formed of age and cohort, it is unsurprising that the model selection criteria choose the more parsimonious option. By arbitrarily adding the quadratic cohort effect, and removing the cohort and period dummy variables from the model, they are testing apples against oranges—it is this change in the degrees of freedom that makes the difference, not the two dimensions per se. No explanation is offered for this test choice, given that in real substantive research, one would not know the true DGP that produced the data.

Reither et al. (2015a) go on to question our judgement that the existence of period and cohort random effects in the DGP meant that model selection that does not find APC effects were incorrect. They argue that, because these random effects are small, it is reasonable for the model to not pick them up as statistically significant. For this reason, the AIC/BIC statistic would not choose the full APC model. This is again true; however it follows from this reasoning that, were these random effects’ variances bigger, or the sample size bigger, these non-linear variations would be picked up by the model as significant. In these instances, the HAPC model would be selected on the basis of the fit statistics. The linear dependency of APC in the model does not disappear because there is more data, or because the noise around those linear trends is greater. As such, the results that would be found may be incorrect, because, whilst non-linear APC effects do exist in the DGP (suggesting the HAPC model), linear effects also exist that could still be radically mis-apportioned. indeed, this is the case in the simulations in Bell and Jones (2014a) in which the HAPC model also performed poorly, yet model fit statistics of the sort used by Reither et al. (2015a) suggest the full APC model is preferable.

The point, here, is that Reither et al. (2015a) do not appreciate that the presence of non-linear effects in a data generating process does not mean that there aren’t *also* near-linear effects, and it does not mean that those near-linear effects could not be themselves confounded, producing results that are incorrect and highly misleading. Model selection criteria can only tell us about non-linear effects (because the linear components can be apportioned in an infinite number of different ways), and as such, they should not be used to judge whether the HAPC model should be used.

### Visual tests of whether the HAPC model should be used

In their latest rejoinder, Reither et al. (2015a) introduce new tests for whether the HAPC model should be used: a visual inspection of (a) the raw APC descriptive trends, and (b) the modelled APC trends produced by the HAPC model (unfortunately, these are rather confusingly conflated in the article).

It is unclear exactly why these would be relevant in deciding whether the HAPC is appropriate. Regarding raw descriptive trends, it will often be the case that trends appear similar. For example the mirror image between period and cohort trends will often be present when there has been change over time. The similarities between age and cohort trends are also unsurprising since older people in the sample are generally born earlier. Thus, we agree that the HAPC model should not be used in these circumstances; however we do not agree that differences in the descriptive APC plots (such as those in Fig. 3 of Reither et al. 2009) are in any way a sufficient test for whether the HAPC model produces valid inference.

The reason for this matches our concerns about using model fit statistics—non-matching APC descriptive plots imply the existence of non-linear APC effects in the DGP, but do not imply that there aren’t *also* linear or near-linear effects in the DGP as well. The argument presented by Reither et al. (2015a) implies that the existence of non-linear effects in a DGP mean that near-linear APC effects are no longer problematic; this is simply incorrect. Such non-linearities simply obscure the near-linear effects in the descriptive plots—they may still be there, and they can still be apportioned in an infinite number of ways between age, period and cohort. Visual plots do not provide a way forward for they are indeterminate in their diagnosis.

Reither et al. (2015a) similarly argue that similarities in modelled outcomes make the HAPC model unsuitable. But the logic behind this is not clear to us. Each trend is, supposedly, controlled for the other trends, so any similarities in trends are not to do with the collinearity between the variables. It is also unclear what counts as ‘similar’. If age and period both show a general upward trend, are they too similar? How much curvature or random variation is needed in each trend before the HAPC model should be allowed? Such questions quickly reveal the flaws in the logic of these criteria, revealing, once again, that these arguments do not consider the possibility that linear and non-linear effects might be present in the same DGP, and that the presence of the latter does not imply that the former is benign. Reither et al. (2015a) also correctly point out a typo in the code that produced our graph—a zero where a nine should be. However, as we argue above, we do not see the relevance of this to their argument.

In sum: the new criteria proposed by Reither et al. (2015a) are arbitrary, and readers only need to look to previous work (Bell and Jones 2014a) and this paper for simulated data where these criteria pass and yet the HAPC model fails to produce sensible and ‘correct’ results.

## Conclusions

If it were the case that there was genuine social process that was driving a certain age, period and cohort combination, we would expect to find it regardless of the data structure at hand. However, the results here show the data structure has a substantial and sometimes determining influence on the results that are produced.

To justify how these results could have occurred whilst still seeing the HAPC model as an appropriate one, one would have to do some fairly dramatic mental gymnastics. First, you would need to argue that the consistency that the real-life results present with simulations has occurred by chance. Second, you would need to come up with a reason why the HAPC model is inappropriate in the data scenarios used here. Third, you would need to argue that there are somehow real differences within these samples that are driving these results to match the simulations (even though the samples heavily overlap with each other). We do not consider any of these arguments to be plausible or defensible. The only sensible explanation in our view is that a statistical artefact of the data structure is driving the results that are found. This explanation also explains those results found by the HAPC models that go against the prevailing academic wisdom (for example Dassonneville 2013, who finds no cohort effects in electoral volatility, despite the literature suggesting such effects should be important).

It is worth concluding by considering some of the arguments that have been made in favour of the HAPC model by its proponents in the literature to date:

- It works because of the inclusion of the age squared term (Yang and Land 2006, p. 84),
- It works because age is treated on a different hierarchical level to periods and cohorts (Yang and Land 2013a, p. 191),
- It works because linear effects do not exist in the real world (Reither et al. 2015b),
- It only works when a model fit statistic says there is non-linear variation in all three of APC (Reither et al. 2015a),
- It only works when raw descriptive plots of APC look dissimilar (Reither et al. 2015a),
- It only works when model predicted plots of APC look similar (Reither et al. 2015a),
- It only works on non-simulated data (Reither et al. 2015a).

In this article, and in our previous contributions, we have provided evidence that every one of these suggestions is flawed, and that the model is never able to reliably apportion near linear APC trends, regardless of what other non-linear processes are present in the DGP. It seems to us that proponents of the HAPC model do not really know why their model works (because it often does not), and are searching under stones to find reasons to justify the continued use of the model.

A final note on replication: it can be argued that one solution to these problems is replication, and it is true that many papers using the HAPC and IE may have been rejected if reviewers had the possibility to replicate results with different methods to test their robustness. We agree (indeed, replication files for this paper are available as online supplementary files for this reason). However successful replication is a necessary but not sufficient condition for HAPC analyses to be robust. Often the implicit identification strategies of different models will be similar or have similar results, and so a second model may often be wrong in the same direction and magnitude as the first.

So, what should likely disheartened reseachers wanting to find independent APC effects do in the absence of a magical solution to the identification problem? For us there are two options. First, authors could choose to remove any linear effects from analyses and focus on patterns of non-linear effects. Such an approach does not solve the identification problem; linear effects cannot be assumed on the basis of these non-linear effects. However often the non-linear effects are in themselves interesting and worthy of publication, so long as the limits of this are made clear (Chauvel and Schroder 2014; Chauvel et al. 2016). Second, and as we have suggested before, researchers should use theory to justify constraints to APC models such as the HAPC model, and those constraints and the reasoning behind them should be stated transparently and explicitly. This might involve a belief that a particular trend will take a certain value, or a view that a certain combination of APC is the most plausible of those possible given the data (Bell 2014; Bell and Jones 2014b, 2015a; Fosse and Winship 2016).

It is worth noting once again that none of this means the HAPC model should be entirely abandoned. The model structure is intuitive and, when constraints are applied that are appropriate and theoretically driven, rather than arbitrary, hidden and statistically driven, the model can produce interesting and important results. However when the model is applied as a mechanical routinized solution that ‘completely avoids’ to the identification problem, dangerously misleading results can be found. Applied researchers should take note.

## Electronic supplementary material

Below is the link to the electronic supplementary material.

Supplementary material 1 (DO 7 kb)

Supplementary material 2 (DO 18 kb)

Supplementary material 3 (DO 8 kb)

Supplementary material 4 (DO 3 kb)

Supplementary material 5 (DO 27 kb)
